# Robotic-assisted bronchoscopic localization for small pulmonary nodules: a novel approach to minimally invasive surgery

**DOI:** 10.3389/fsurg.2025.1641868

**Published:** 2025-09-19

**Authors:** Qiduo Yu, Haoshuai Yang, Jingyu Chen, Fanjia Kong, Jin Zhang, Zhoujunyi Tian, Zhenrong Zhang, Chaoyang Liang

**Affiliations:** Department of Thoracic Surgery, China-Japan Friendship Hospital, Beijing, China

**Keywords:** robotic bronchoscopy, pulmonary nodules, intraoperative localization, lung segmentectomy, lung cancer

## Abstract

**Background:**

Precise intraoperative localization of small pulmonary nodules is crucial for minimally invasive lung surgery. Robotic bronchoscopy, combining electromagnetic navigation and fluorescence marking, addresses limitations of traditional methods.

**Methods:**

This feasibility study included 10 patients (mean age 58) with ground-glass/partially solid nodules (mean diameter 1.42 cm). Using the Monarch® robotic system, nodules were intraoperatively marked with fluorescent dye (indocyanine green/methylene blue). Segmentectomy (4) or wedge resection (6) was performed, with lobectomy added if needed.

**Results:**

All nodules were successfully localized (mean time 16.9 min) without complications. Resected specimens confirmed central nodule placement. Pathology identified primary lung cancer in all cases: 1 adenocarcinoma *in situ*, 3 microinvasive, and 6 invasive. Lobectomy was avoided in 4 segmentectomy cases but required in 5/6 wedge resections.

**Conclusion:**

Robotic bronchoscopic localization enables safe, precise intraoperative marking, minimizing healthy tissue resection. This pilot study supports its clinical potential, warranting larger trials for validation.

## Introduction

Pulmonary nodules (PNs), defined as round or irregular lesions in the lungs with a diameter of 3 cm or less, are increasingly being detected due to advancements in low-dose computed tomography (LDCT) technology (1). Accurate localization of such nodules is crucial for ensuring the safety and accuracy of video-assisted thoracoscopic surgery (VATS), the preferred treatment method for high-risk pulmonary nodules (2). The difficulty in localizing these nodules intraoperatively increases the risk of open surgery conversion and positive surgical margins and increases the complexity of lung segmentectomy and wedge resection (3). Therefore, precise nodule localization is a critical technical aspect of surgery, ensuring safe margins and minimizing the removal of normal lung tissue.

Two primary nonsurgical biopsy methods for pulmonary nodules are percutaneous needle biopsy (PTNB) and transbronchial lung biopsy (TBLB) (4, 5). Owing to its high sensitivity and specificity, PTNB is particularly suitable for larger solid nodules but is associated with a greater risk of complications, such as pneumothorax, bleeding, and needle tract seeding (6). TBLB utilizes various guidance technologies, such as x-ray fluoroscopy, cone-beam CT (CBCT), and ultrathin bronchoscopes, to accurately reach and confirm the lesion's location with tools such as radial endobronchial ultrasound probes (r-EBUS) and endobronchial ultrasound-guided sheaths (EBUS-GS) (7). While TBLB has a lower risk of complications, it requires high levels of technical skill, specialized equipment, and trained physicians, and the procedure can be time-consuming, particularly for very small or deep nodules (4, 8).

In recent years, robotic bronchoscopy technology has emerged as a novel approach for deep lung lesion navigation (9–12). This technology integrates optical recognition, dynamic electromagnetic guidance, and endoscope motion for precise control, enhancing the diagnostic capability for peripheral lung lesions. Studies have shown that robotic-assisted bronchoscopic navigation systems can be used to biopsy lung nodules safely and accurately, especially in areas that are difficult to reach with conventional methods (10, 11). Robotic bronchoscopic localization techniques offer increased localization precision, reduced complication risks, and the potential to overcome the limitations of traditional methods in localizing deep or inaccessible nodules (13, 14). By integrating various imaging technologies, robotic systems can provide comprehensive navigation information for surgical procedures, enhancing their safety and efficacy.

This study aims to explore the uncharted field of robotic bronchoscopic localization technology. By including patients with small pulmonary nodules who are candidates for lung segmentectomy and comparing intraoperative robotic bronchoscopic localization with preoperative percutaneous localization, we aimed to investigate the feasibility and clinical application value of robotic bronchoscopic localization technology, providing clinical data support for its use in clinical practice. The primary scientific significance of this study lies in addressing the important clinical challenge of improving the localization accuracy rate for patients with small pulmonary nodules, with a focus on simple and safe localization methods to provide high-level evidence for clinical decision-making.

## Materials and methods

### Ethics approval

This study was performed in accordance with the principles of the Declaration of Helsinki. Approval was granted by the Ethics Committee of China-Japan Friendship Hospital (No. 2022-KY-127).

### Patient selection

From September 2024 to December 2024, the selected patients were hospitalized in the Thoracic Surgery Department of China-Japan Friendship Hospital for surgical treatment due to pulmonary ground glass or partial solid ground glass nodules. The patients met the following inclusion criteria: the lesion was a single pure ground glass nodule or partially solid ground glass nodule with a length of less than 3 cm. On the basis of the influence of CT, the nature of the nodule is likely early-stage lung cancer. During the operation, sublobectomy (lung segment or wedge resection) is needed first. The nature of the lesion is determined by intraoperative frozen pathology, and then the surgeon determines whether to continue with lobectomy. Therefore, auxiliary localization is needed for local resection.

Patients who met any of the following exclusion criteria were excluded: (1) The patient has medical contraindications for bronchoscopy or anaesthesia (severe arrhythmia, severe hypertension, severe cardiopulmonary dysfunction, myocardial infarction, unstable angina, etc.); (2) The patient has uncontrollable or irreversible coagulation dysfunction and bleeding tendencies (such as coagulation disorders, uremia, active massive hemoptysis, etc.); (3) Patients who wear or implant medical devices that interfere with electromagnetic navigation, including but not limited to pacemakers; (4) Pregnant or lactating female patients.

### Operation method

**Figure 1:**
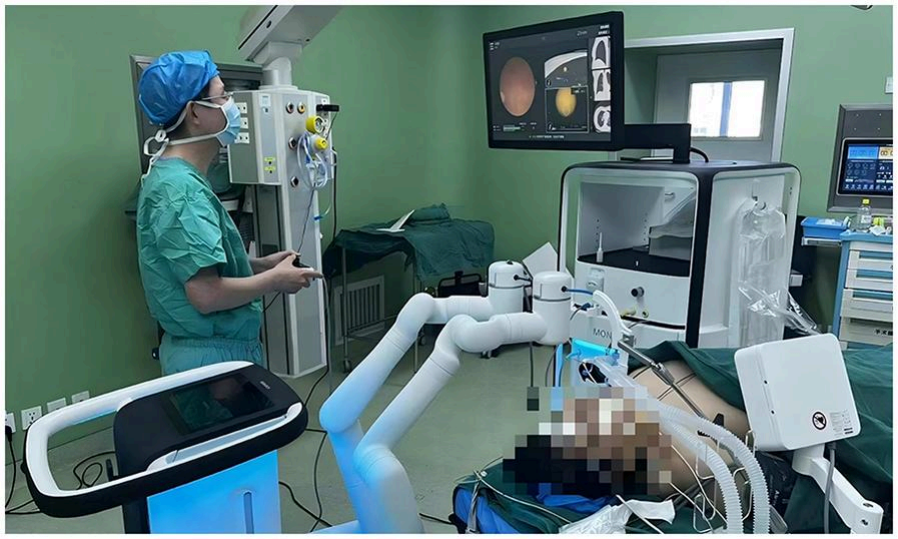
Thoracic surgeon operates bronchoscope robot for lesion localization.

After the patient enters the operating room, under general anaesthesia, a thoracic surgeon performs fiberoptic bronchoscopy to rule out airway diseases and aspirate airway secretions. Then, the thoracic surgeon performs a robot-assisted bronchoscopy examination to reach the nodule position at the end of the bronchoscope (Figure 1). A matching puncture needle was used to annotate 2.5 ml of mixed fluorescence staining agent (2 ml of indocyanine green and 0.5 ml of methylene blue) at the nodule location. Indocyanine green is a fluorescent chromogenic agent that can be detected via fluorescence thoracoscopy through the lung parenchyma during surgery. After the injection of methylene blue, airway blue staining can be observed via bronchoscopy to confirm that the dye has indeed been injected into the target site. The robot-assisted bronchoscope system was then removed. Two thoracic surgeons performed video-assisted thoracic surgery. During the operation, the marked area was displayed through fluorescence thoracoscopy, and sublobectomy (segmental resection or wedge resection) was performed first. The excised specimen was sent for frozen pathological examination. On the basis of the pathology results, the thoracic surgeon determines whether further lobectomy and lymph node dissection are necessary.

## Results

### Patient characteristics

A total of 10 patients were selected, including 4 males and 6 females. Supplementary Table S1 shows the general characteristics, lesion locations, localization processes, and surgical approaches of all the patients. The age range was 41–86 years, with an average age of 58 ± 13 years. The average longest diameter of the lesions was 1.42 ± 0.52 cm. The nodules were located in the upper lobe of the right lung in 7 patients, the middle lobe of the right lung in 1 patient, and the lower lobe of the right lung in 2 patients. The average duration required for localization was 16.9 ± 13 min.

### Localization procedure outcomes and pathology finding

The surgical plan for each patient was determined through consultations and discussions by a team of multiple experts. Among them, four patients were scheduled for segmentectomy, which required intraoperative localization to ensure adequate surgical margins. The other six patients required preoperative localization to precisely identify the lesion sites for wedge resection, with the decision on whether to proceed with lobectomy based on intraoperative pathological findings. Four patients underwent segmentectomy as expected. Among the 6 patients who underwent wedge resection, 1 patient did not require lobectomy, while 5 patients continued with lobectomy during the same period.

During the localization process, there were no complications, such as bleeding, pneumothorax, or color reagent allergy. All the nodules are located in the central position within the excised specimen. Intraoperative frozen pathology reports revealed that all 10 patients had primary lung cancer, including 1 patient with adenocarcinoma *in situ*, 3 patients with microinvasive adenocarcinoma, and 6 patients with invasive adenocarcinoma. The characteristics of the localization and procedure are summarized in Table 1.

**Table 1:** Characteristics of the patients, localization and procedure.

Patients character	Description
Age (years)	58 ± 13 (41–86)
Female/male	6/4
Location	
Light upper lobe	7 (70%)
Right middle lobe	1 (10%)
Right lower lobe	2 (20%)
Lesion size (cm)	1.42 ± 0.52
Localization duration (min)	16.9 ± 6.0
Localization success rate	100%
Complications during the localization process	0%
Frozen pathology	
Adenocarcinoma *in situ*	1 (10%)
Microinvasive adenocarcinoma	3 (30%)
Invasive adenocarcinoma	6 (60%)
Procedure	
Segmentectomy	4 (40%)
Wedge resection only	1 (10%)
Wedge resection then lobectomy	5 (50%)

### Representative cases

**Figure 2:**
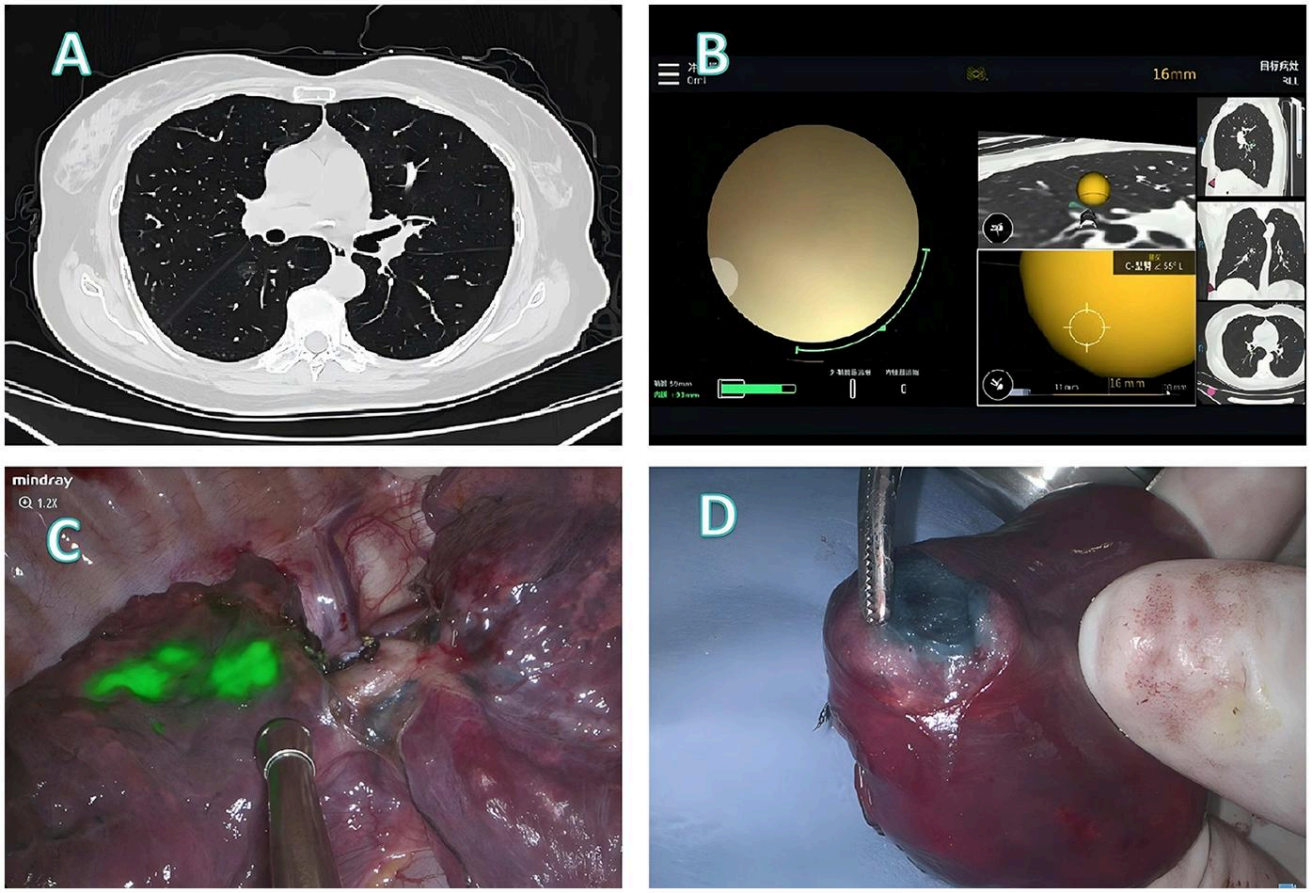
Localization and surgical procedure process for a 61-years-old patient. **(A)** CT scan shows a ground glass nodule in the lower lobe of the right lung, located near the pleura on the lung fissure side. **(B)** Localization process with the assistance of a robotic bronchoscope. **(C)** Under fluorescence thoracoscopy, the lesion location was visualized on the pleura of the lung fissure during surgery. **(D)** A segmentectomy (RS6b+c) is performed, with the lesion located at the center of the excised specimen.

**Figure 3:**
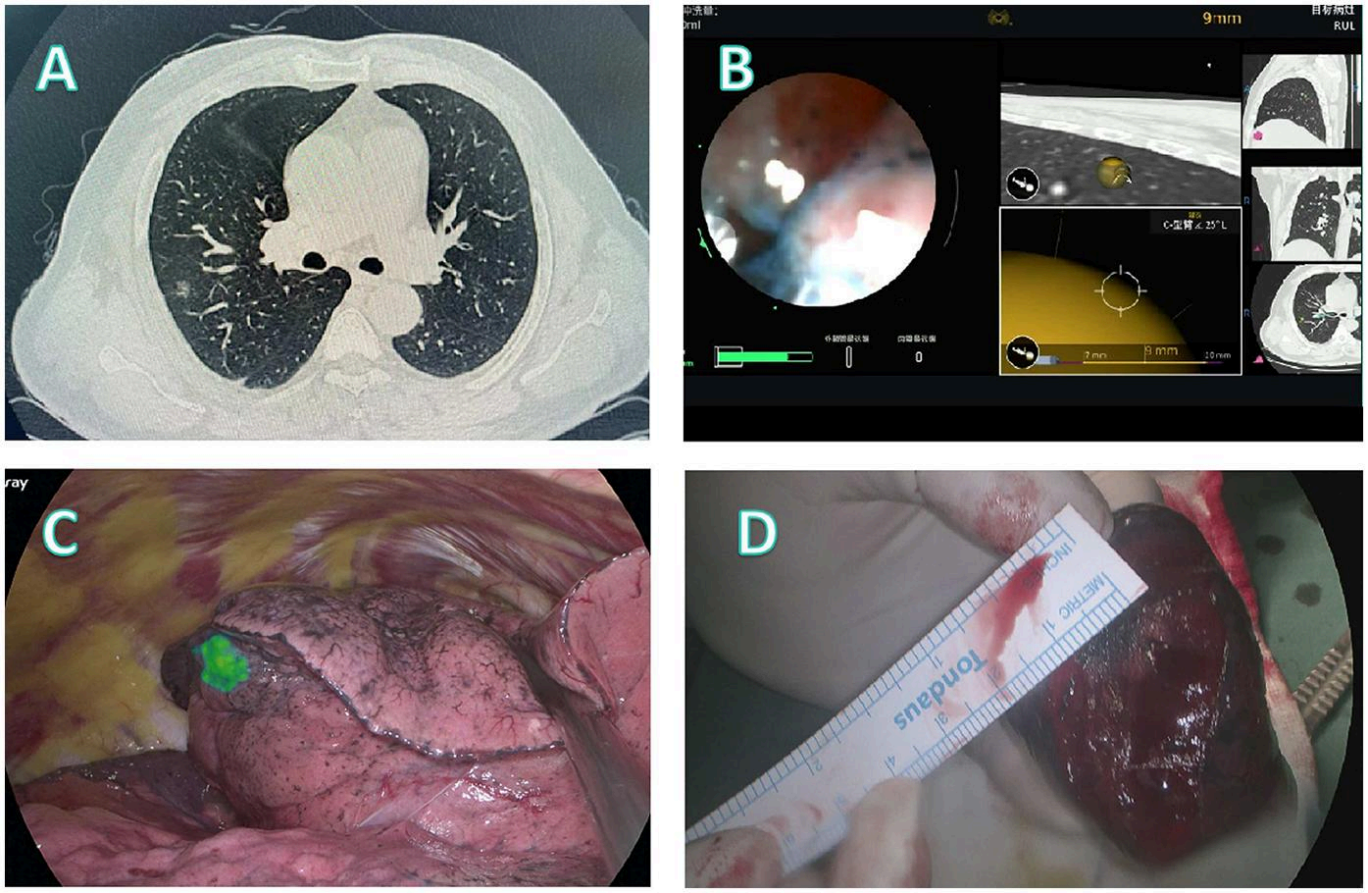
Localization and surgical procedure process for a 52-years-old patient. **(A)** CT scan shows a partial solid ground glass nodule in the upper lobe of the right lung. **(B)** Localization process with the assistance of a robotic bronchoscope. **(C)** Under fluorescence thoracoscopy, the lesion location was visualized on the visceral pleura of the lung fissure during surgery. **(D)** A segmentectomy (RS3a) is performed, with the lesion located at the center of the excised specimen.

**Figure 4:**
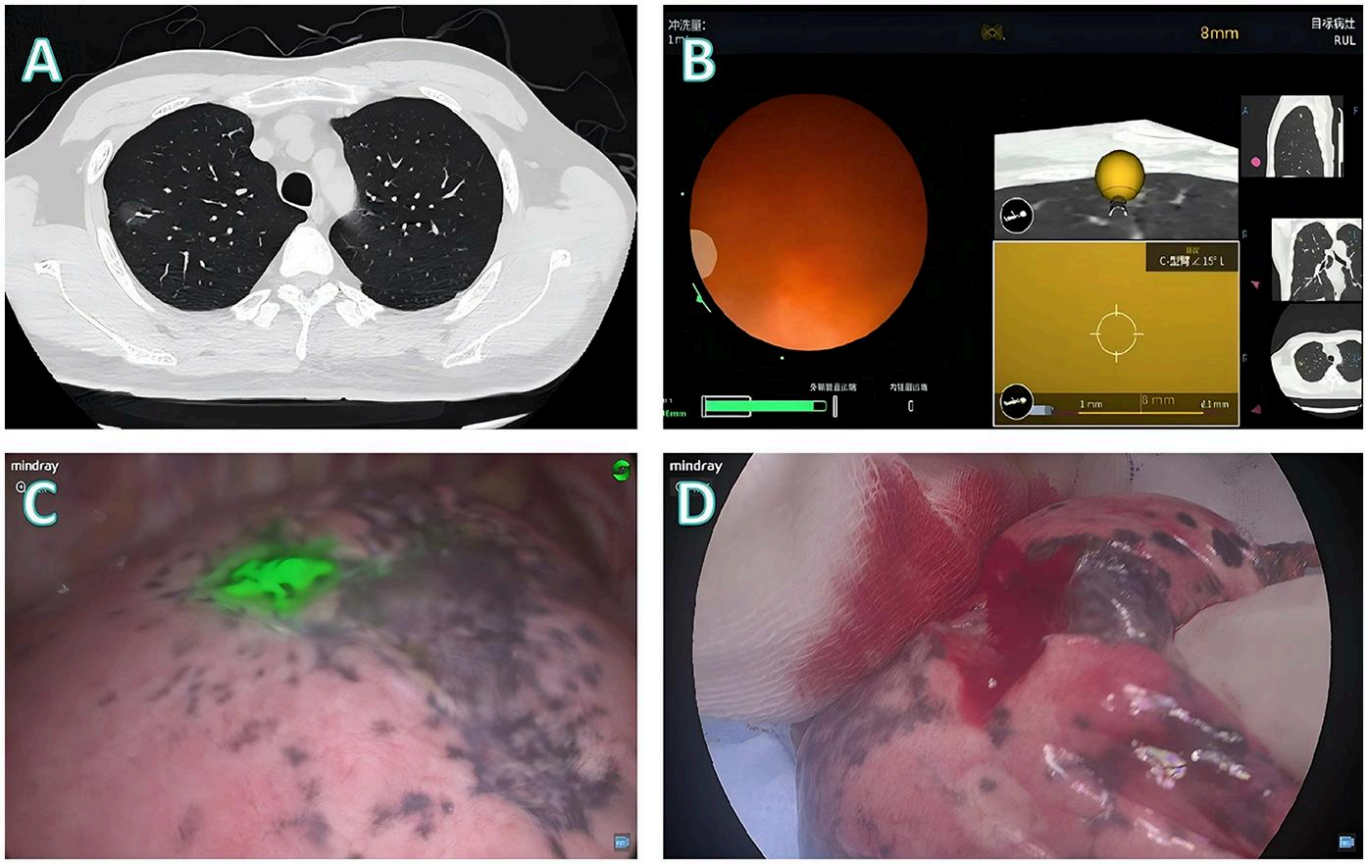
Localization and surgical procedure process for a 41-years-old patient. **(A)** CT scan shows a partial solid ground glass nodule in the upper lobe of the right lung. **(B)** Localization process with the assistance of a robotic bronchoscope. **(C)** Under fluorescence thoracoscopy, the lesion location was visualized on the visceral pleura during surgery. **(D)** Wedge resection is performed, with the lesion located at the center of the excised specimen.

Three representative cases from the dataset were selected to demonstrate our localization and surgical approaches, as illustrated in Figures 2–4.

**Patient 1** (Figure 2): A 61-year-old female presented with a ground glass nodule in the right lower lobe adjacent to the fissural pleura (Figure 2A). Conventional percutaneous access was deemed unsuitable because the deep-seated location required traversal of substantial normal parenchyma. Robotic bronchoscopy enabled precise delivery of the fluorescence staining cocktail to the target lesion (Figure 2B). Intraoperative fluorescence imaging successfully demarcated the fissural pleural projection of the lesion (Figure 2C), guiding an anatomical RS6b+c segmentectomy. Histopathological examination of the centrally located specimen confirmed microinvasive adenocarcinoma (Figure 2D).

**Patient 2** (Figure 3): In this 52-year-old male patient, CT imaging revealed a partially solid nodule in the right upper lobe (Figure 3A). Scapular obstruction precluded safe percutaneous access, prompting robotic bronchoscopic administration of fluorescent markers (Figure 3B). Fluorescence thoracoscopy clearly delineated the lesion on the visceral fissural pleura (Figure 3C), facilitating targeted RS3a segmentectomy. The resected specimen demonstrated centrally located invasive adenocarcinoma with clear margins (Figure 3D).

**Patient 3** (Figure 4): A 41-year-old male presented with a mixed-density nodule in the right upper lobe (Figure 4A). Given that the scapular shadow impedes the use of percutaneous approaches, robotic bronchoscopic fluorescence marking was performed (Figure 4B). Intraoperative imaging revealed that the lesion was localized to the visceral pleura (Figure 4C), initially indicating wedge resection. Frozen section analysis revealed that invasive adenocarcinoma with micropapillary components necessitated conversion to lobectomy, with final pathology confirming complete tumor excision (Figure 4D).

## Discussion

With the increasing use of CT for lung cancer screening, the need for precise diagnostics and minimally invasive treatments for pulmonary nodules is increasing (15). Judging on the basis of preoperative CT examination, it is difficult for clinical surgeons to locate it through visual observation or touching during surgery. The thoracic surgeon believes that preoperative localization is necessary to perform wedge resection of the lesion on the basis of the location for frozen pathological examination or to determine the range of the lung segment resection margin. In recent years, several advanced robotic bronchoscopy systems have been developed and put into use to increase the diagnostic accuracy and safety of small pulmonary nodules. Robotic bronchoscopy-guided marking has shown promise in safely and effectively localizing these lesions during surgery, potentially reducing the need for more extensive procedures such as lobectomies (16, 17). This study explored the feasibility of using a robotic bronchoscopic system for intraoperative localization. Preliminary results suggest that reliance on a robotic bronchoscopic system can achieve relatively accurate localization with good safety, indicating its potential for clinical application.

The Monarch® bronchoscopic robot system used in this study features two independent robotic arms capable of precise manipulation (18). Previous studies have demonstrated that the robotic bronchoscopic system offers significant benefits in terms of nodule navigation and biopsy, enhancing the diagnostic accuracy of small pulmonary nodules and minimizing complications, thus proving to be both practical and safe. In the REACH validation study (19), the bronchoscopic robot demonstrated superior navigational capabilities compared with conventional bronchoscopes in all lung segments, especially in bronchi with larger angles. In the ACCESS study (20), the system achieved a high diagnostic rate of 97% when performing biopsies on 67 artificial tumors implanted in 8 cadavers, indicating a high level of accuracy. In the feasibility cohort study (21), the bronchoscopic robot achieved a biopsy sample acquisition rate of 93% in 15 patients without any severe complications. The first large multicenter retrospective study (22) analysed the performance of a bronchoscopic robot combined with x-ray fluoroscopy in 167 lung nodule navigation biopsies across 165 patients. The study revealed that 70.7% of lesions were located in the peripheral third of the lung, with 64% exhibiting bronchial signs.

In our study, the surgical approach for all 10 patients was determined by multidisciplinary discussion. Four patients underwent direct segmentectomy due to small nodules (less than 1 cm; solid component <50%) where sublobar resection was adequate. However, segmentectomy was chosen over wedge resection because the nodules were adjacent to segmental vessels and bronchi, thus reducing the risk of injury. The other six patients, with larger and more solid nodules, initially underwent wedge resection to obtain frozen sections for further decision. One case of minimally invasive adenocarcinoma required no further resection. The other five, with invasive adenocarcinoma (including micropapillary or mucinous subtypes), proceeded to lobectomy.

During the operational process, we identified significant technological advantages of the robotic bronchoscope. The bronchoscope can be advanced further into the peripheral bronchi and navigate complexly through small and tortuous bronchial paths. Even for lesions located beyond visible airways, we advanced the scope through the parenchyma under navigation guidance to approach the target. When within 2 cm of the lesion, we used a matched needle to puncture and inject dye. Thus, dye marking was feasible regardless of the bronchus sign. Upon CT review, 3 cases had a bronchus sign while 7 did not—all were successfully marked. Compared with the traditional electromagnetic navigation bronchoscope (ENB), the robotic bronchoscope offers operators a greater number of independently controllable components, thereby enhancing operational flexibility. Traditional bronchoscopy may struggle to access apical or diaphragmatic nodules due to mobility and navigation limitations, the Monarch system's dual-stage articulating design allows stable advancement into peripheral airways. Among the 10 nodules, 6 were located in the outer third, 2 in the middle third, and 2 in the inner third of the lung. Dye was visible on the visceral pleura for outer third lesions. For the middle and inner third nodules, the dye diffused toward the nearest fissural or mediastinal pleural surface, achieving satisfactory localization. Additionally, the robotic bronchoscope provides an excellent field of vision, and we found that controlling the advancement and bending of the bronchoscope through a controller is highly intuitive. This feature results in a smoother learning curve, allowing young physicians to rapidly develop their skills. Previously, without robotic control, operators often had to raise their left arm high above their shoulder and contort it into awkward angles to control the direction of traditional bronchoscopes; the joystick operation has eliminated these inconveniences.

At the same time, we also summarized the experience of anesthesia during the operation process. Basal lung atlectasis is easily happed under general anesthesia, especially in supine position. To prevent atelectasis in the lower lobe basal segments of patients in the supine position, which could lead to nodule localization failure, comprehensive measures are taken during surgery. These include immediately suctioning secretions with a bronchoscope after intubation to ensure bronchial patency, maintaining continuous positive pressure ventilation with an appropriate PEEP level to prevent alveolar collapse, monitoring airway pressure by the anesthesiologist with prompt bronchoscopic suctioning and recruitment maneuvers if a sudden pressure increase occurs, performing key steps such as puncture or dye injection during an inflation hold at the end of inspiration, and minimizing the duration of the entire localization procedure.

Traditional robotic bronchoscopy localization requires accurate images provided by cone-beam computed tomography (CBCT), which places high demands on the size and setup of the operating room (23). Additionally, hybrid operating rooms necessitate substantial initial setup costs and subsequent maintenance expenses, not to mention the cost of the robotic platform itself. This study attempted localization by relying solely on preoperative information and a robotic system, and the results demonstrated that this procedure is highly feasible and safe.

However, the study's limitations, including its single-center design, small patient cohort, and lack of long-term safety evaluation, highlight the need for further research. Future studies should aim to validate these findings in larger, multicenter trials and assess the long-term outcomes and cost‒benefit analysis of robotic bronchoscopic localization (24). Meanwhile, constrained by the radiation protection conditions in the operating room, we did not use CBCT for verification, which made it impossible to reconfirm the accuracy of the positioning or conduct comparisons before the surgery.

This study demonstrates the feasibility of robotic bronchoscopic localization for the intraoperative marking of small pulmonary nodules, offering a precise and minimally invasive alternative to traditional methods. Using advanced robotic systems, the procedure achieved accurate nodule localization with minimal complications, enhancing the safety and efficacy of lung segmentectomy and wedge resection. The integration of fluorescence and dye marking further improved accuracy, reducing the need for extensive surgeries. The intuitive operation and superior navigation capabilities of the robotic system suggest a smoother learning curve for surgeons. These findings support the clinical adoption of robotic bronchoscopic localization as a valuable tool for improving pulmonary nodule surgery outcomes.

## Data Availability

The original contributions presented in the study are included in the article/Supplementary Material, further inquiries can be directed to the corresponding authors.
