# Supplementary material for: Robotic-assisted bronchoscopic localization for small pulmonary nodules: a novel approach to minimally invasive surgery
Source: Front Surg. 2025 Sep 19;12:1641868. doi: 10.3389/fsurg.2025.1641868 (PMC12491215; doi:10.3389/fsurg.2025.1641868)
Supplement: Supplementary file 1 [file Table1.docx]

| Case | Age | Female/male | Location | Lesion size (cm) | purpose | Positioning duration (min) | Localization success | Localization complication | Procedure | frozen pathology | continued lobectomy |
| --- | --- | --- | --- | --- | --- | --- | --- | --- | --- | --- | --- |
| 1 | 54 | M | RUL | 0.9 | Determine cutting edge | 30 | + | - | Segmentectomy RS1 | AIS | - |
| 2 | 41 | F | RLL | 0.6 | Determine cutting edge | 15 | + | - | Segmentectomy RS6 | MIA | - |
| 3 | 61 | F | RLL | 1.2 | Determine cutting edge | 21 | + | - | Segmentectomy RS6_b+c_ | MIA | - |
| 4 | 86 | F | RUL | 2.2 | Positioning for wedge resection | 14 | + | - | wedge resection | IAC | + |
| 5 | 58 | M | RUL | 1.1 | Positioning for wedge resection | 12 | + | - | wedge resection | MIA | - |
| 6 | 52 | M | RUL | 1.1 | Determine cutting edge | 8 | + | - | Segmentectomy RS3_a_ | IAC | - |
| 7 | 41 | M | RUL | 2.0 | Positioning for wedge resection | 17 | + | - | wedge resection | IAC | + |
| 8 | 59 | F | RUL | 1.9 | Positioning for wedge resection | 15 | + | - | wedge resection | IAC | + |
| 9 | 68 | F | RUL | 1.7 | Positioning for wedge resection | 21 | + | - | wedge resection | IAC | + |
| 10 | 63 | F | RML | 1.5 | Positioning for wedge resection | 16 | + | - | wedge resection | IAC | + |

Supplementary Table1. Information of all cases. RUL, right upper lobe; RLL, right lower lobe; RML, right middle lobe; AIS, Adenocarcinoma in situ; MIA, Microinvasive adenocarcinoma; IAC Invasive adenocarcinoma
